# Response to the Letter-to-the-Editor: The Search for the Ideal Female Breast–A Nationally Representative United-States-Census Study

**DOI:** 10.1007/s00266-022-02849-z

**Published:** 2022-07-05

**Authors:** Christoph Wallner, Marcus Lehnhardt, Björn Behr

**Affiliations:** Department of Plastic Surgery, BG University Hospital Bergmannsheil, Ruhr University Bochum, Bürkle-de-la-Camp Platz 1, 44789 Bochum, Germany

*Level of Evidence V* This journal requires that authors assign a level of evidence to each article. For a full description of these Evidence-Based Medicine ratings, please refer to the Table of Contents or the online Instructions to Authors www.springer.com/00266.

We are pleased about the unexpectedly large positive feedback to the paper we published [1]. The scale of the survey is novel and covers a cross section of the US population. Thereby, the online survey enabled us to study a representative proportion. As in all survey forms, the possibility of bias due to the survey medium is embedded. However, studies clearly demonstrate the superiority of this new and very effective form of study [2, 3]. The survey platform allows the results to be assigned to the respective region. Hence, on the one hand, we can reproduce that the study distribution within the USA roughly corresponds to population density; on the other hand, we were able to carry out an analysis about the distribution, but did not find any significant differences. In a study population with 1000 participants, we indeed identified ethnic differences, but a further regional subdivision would no longer have been statistically meaningful.

We also see the importance of the studies by Mallucci et al., who frankly were the initial inspiration. They started with an initial evaluation of breast morphometry, and we extensively discussed the results of these studies in the context of our study. As stated before, we perceived the opportunity for improvement of the methodology of such a study, including the statistics. These circumstances result in the apparently clear outcomes across all ethnic groups in the quoted study [4]. One core statement of our work, which can be found both in the illustrations and in the discussion, is tendencies given by the survey. We have not seen outliers that clearly outweigh a preference other than the discussed article [4]. Only a tendency can be derived from our study since all parameters correspond to a Gaussian distribution in terms of their preference.

You already mention the methodological inadequacy of the other work you quoted: “…100 topless cover models with subjectively appealing breasts…”. These models were selected by the magazine officials of *The Sun* for a certain target group [5]. If anything, these images only reflect preferences of the target group. However, from these studies, we were able to draw important conclusions about the methodology and used them in the design.

We affirm that our study only considered a section of the relevant breast dimensions. The study concentrated on factors most discussed in the community, which represent modifiable parameters for breast augmentation, reduction and reconstruction. In addition, we see a great advantage of our study in the fact that each parameter is considered individually in every picture set. Subsequently, we succeeded in achieving harmonic breast representations based on changing individual parameters, which has not been published so far. To our knowledge, the abundance of various breast-shaping factors as presented in our study could not be reproduced in any previously published study. Here, schematic and anatomically undetailed representations were utilized. Moreover, images were morphed without the overall picture adapted accordingly. Most of the studies published to date consider a maximum of two factors. Therefore, we consider our study as a comprehensive overview about measurable breast aesthetics.

We completely agree that the breast should be harmonious in its entirety, but some factors represent controllable variables in breast surgery, which should be determined on a broad scientific basis. In our opinion, this scientific approach has so far been missing. We continue to see that certain parameters found in our study not only reflects public opinion, but might also be implemented in surgical practice. Based on the sound census data, it is hard to perpetuate the 45:55 distribution as a dogma in breast surgery. When approaching questions related to breast aesthetic, we propose to also consider scientific methods utilized by social psychologists and anthropologists [6, 7].
